# 5MinuteConsult

**DOI:** 10.5195/jmla.2018.406

**Published:** 2018-04-01

**Authors:** Ivan ArmandoPortillo

**Affiliations:** Leatherby Libraries, Chapman University, Orange, CA

5MinuteConsult is an online resource that is designed to help health care professionals and clinical students make evidence-based decisions by providing information that is needed at the point-of-care [1]. Originally developed in 2008, 5MinuteConsult was meant to be the online version of the book *5-Minute Clinical Consult,* which is now in its twenty-sixth edition. The physical title, released annually with updated content, provides quick access to information on common and important conditions and symptoms. The initial goal of the print version was for a physician to be able to locate a specific topic in less than thirty seconds, which is no longer realistic due to the increase in pages and physical size [2].

Initially included with the purchase of the print copy, the digital version is now a stand-alone product that is available by subscription. It is intended to offer easier access and additional content. The website now offers responsive design for mobile devices, procedure videos, and continuing medical education (CME)/continuing education (CE) credits, as well as algorithms and topics that are not available in the print editions.

5MinuteConsult is also available as an app for iOS and Android devices, but at the time of writing, these were only available for an additional cost.

## OVERVIEW

5MinuteConsult has a very simple layout with just a search bar and five tabs at the top of the page. Speed appears to be a key feature for the website: the “About” section states that 5MinuteConsult “is the fastest resource to obtain the most likely diagnosis, treatment, and management for thousands of diseases” [1]. The content is broken down into five sections: Diseases & Conditions, Drugs, For the Patient, Tools, and CME/CE.

While the majority of the content from the physical book can be found in these five tabs, Wolters Kluwer has also integrated several other products from its catalog into the website, including content from *5-Minute Clinical Consult, 5-Minute Pediatric Consult, Rosen & Barkin’s 5-Minute Emergency Medicine Consult, 5-Minute Consult Clinical Companion to Women’s Health, Essential Guide to Primary Care Procedures,* and *A Practical Guide to Joint & Soft Tissue Injection;* drug monographs from Facts & Comparisons; laboratory test interpretation from *Wallach’s Interpretation of Diagnostic Tests*; patient handouts from Lexicomp; and International Classification of Diseases (ICD)-10, ICD-9, and SNOMED codes.

## CONTENT

According to a representative from Ovid, the content in 5minuteConsult is updated quarterly and in conjunction with the release of the newest edition of the title. The information and recommendations that are available on the site are provided by primary care physicians who collaborate with the editorial team of *5-Minute Clinical Consult* [2]. The majority of the content from the physical books is contained in the Diseases & Conditions and Algorithms & Charts sections of the website. The Diseases & Conditions section includes topic pages on over 2,000 diseases and conditions, with several offered in both English and Spanish. Each of these pages contains information broken down into separate sections such as Basics, Diagnosis, Treatment, and Ongoing Care. Certain topics also include references, additional readings, clinical pearls, and related ICD and SNOMED codes.

5MinuteConsult includes a grading system for the evidence found in the Diseases & Conditions section based on the American Academy of Family Physicians Strength of Recommendations Taxonomy (SORT). A grade is only given when a piece of information contains a reference, and the reference is then given a grade of either A (consistent, good-quality patient-oriented evidence), B (inconsistent or limited-quality patient-oriented evidence), or C (consensus, disease-oriented evidence, usual practice, expert opinion, or other evidence) [3]. References are given with full citations and links to abstracts through Ovid Insights. The grading system and links to abstracts through Ovid Insights are only available in the Diseases & Conditions section.

The drug monographs in 5MinuteConsult contain content from Facts & Comparisons, a trusted drug information resource for pharmacists, but critical information from that source is missing. Strength of evidence data has been omitted, along with links to abstracts or a bibliography. The user is left without the article title, journal title, and information about the currency of the monographs. The lack of these features indicates that the drugs section of 5MinuteConsult should not be used as the only drug information resource at an institution.

## TOOLS

5MinuteConsult offers procedures, algorithms, charts, and lab tests in its Tools section. Over 200 procedures are available, some of which include images, figures, tables, and/or videos. Procedures are written by physician specialists and offer bibliographies, with many including links to abstracts, but there is no indication of when the content was last updated.

Nearly 300 diagnosis and treatment algorithms are available in the Tools section. Each algorithm offers a visual method for clinicians to evaluate abnormal symptoms and prioritize treatments [3]. Users will find these algorithms helpful when differentiating symptoms and selecting treatments. The majority of the algorithms and charts offer a full citation and date of last update.

The content found in the more than 450 Lab Tests comes from *Wallach’s Interpretation of Diagnostic Tests.* These tests offer recommendations for when to order the “correct test and how to interpret the results” [4]. Test pages often include tables and suggested reading, along with links to related topics in 5MinuteConsult.

While no advanced search is offered, the persistent search bar is sufficient. Once a keyword search is performed, users can filter results and preview images that are contained in topic pages.

## PATIENT HANDOUTS

5MinuteConsult includes over 3,000 patient education handouts, with the majority available in English and Spanish. Handouts can be customized to include contact information and a message to the patient. Patient handout information is provided by Lexicomp, a drug information resource from Wolters Kluwer [1]. The handouts provide an easy reading level, illustrations, links for patients to find further information, and a review date.

## CONTINUING EDUCATION

5MinuteConsult offers point-of-care CME/CE credits to physicians at no additional charge. Credit is earned by searching for a topic and then answering a questionnaire after reviewing the material. Each submitted questionnaire is worth 0.5 credits, and up to 20 credits may be earned. This point-of-care activity has been reviewed and accepted by the American Academy of Family Physicians and the American Nurses Credentialing Center’s Commission [1].

## CONCLUSION

5MinuteConsult allows clinicians to find information quickly to support a clinical decision. The interface is easy to use, enabling any physician to navigate and gather information quickly. With the additional content provided by some of the most respected resources in Wolters Kluwer’s catalog, this resource offers advantages over the physical book. Because some important features are lacking, clinicians should not use this resource as their only source.
